# *TRPM1* mutations are associated with the complete form of congenital stationary night blindness

**Published:** 2010-03-12

**Authors:** Makoto Nakamura, Rikako Sanuki, Tetsuhiro R. Yasuma, Akishi Onishi, Koji M. Nishiguchi, Chieko Koike, Mikiko Kadowaki, Mineo Kondo, Yozo Miyake, Takahisa Furukawa

**Affiliations:** 1 Department of Ophthalmology, Nagoya University Graduate School of Medicine, Nagoya, Japan; 2 Department of Developmental Biology, Osaka Bioscience Institute, Osaka, Japan; 3 JST, CREST, Suita, Osaka, Japan; 4 JST, PRESTO, Kawaguchi, Saitama, Japan; 5 Department of Orthoptics and Vision Science, Aichi Shukutoku University, Nagoya, Japan

## Abstract

**Purpose:**

To identify human transient receptor potential cation channel, subfamily M, member 1 (*TRPM1)* gene mutations in patients with congenital stationary night blindness (CSNB).

**Methods:**

We analyzed four different Japanese patients with complete CSNB in whom previous molecular examination revealed no mutation in either nyctalopin (*NYX)* or glutamate receptor, metabotropic 6 (*GRM6)*. The ophthalmologic examination included best-corrected visual acuity, refraction, biomicroscopy, ophthalmoscopy, fundus photography, Goldmann kinetic perimetry, color vision tests, and electroretinography (ERG). Exons 2 through 27 and the exon-intron junction regions of human *TRPM1* were sequenced.

**Results:**

Five different mutations in human *TRPM1* were identified. Mutations were present in three unrelated patients with complete CSNB. All three patients were compound heterozygotes. Fundus examination revealed no abnormalities other than myopic changes, and the single bright-flash, mixed rod-cone ERG showed a “negative-type” configuration with a reduced normal a-wave and a significantly reduced b-wave amplitude. Our biochemical and cell biologic analyses suggest that the two identified IVS mutations lead to abnormal TRPM1 protein production, and imply that the two identified missense mutations lead to the mislocalization of the TRPM1 protein in bipolar cells (BCs).

**Conclusions:**

Human *TRPM1* mutations are associated with the complete form of CSNB in Japanese patients, suggesting that TRPM1 plays an essential role in mediating the photoresponse in ON BCs in humans as well as in mice.

## Introduction

The complete form of congenital stationary night blindness (CSNB) is a subtype of Schubert-Bornschein CSNB in which the fundus is essentially normal except for myopic changes [1-5]. From early childhood, patients with complete CSNB lack rod function and experience night blindness. Nystagmus and amblyopia sometimes accompany the other symptoms, and the clinical course is stationary. Best-corrected visual acuity is mildly reduced, and high to moderate myopia is usually found. Electroretinogram (ERG) examinations reveal absent depolarization of neuron by light (ON)-responses (b-wave), and previous extensive physiologic studies indicated that the pathology in complete CSNB lies in the dysfunction of the depolarizing ON bipolar cell (BC). There are two hereditary patterns for complete CSNB: X-linked recessive and autosomal recessive [4].

To date, two genes, the leucine-rich proteoglycan nyctalopin gene (*NYX*)—encoding the glycosylphosphatidylinositol (GPI)-anchored extracellular protein nyctalopin [6,7]—and the *GRM6* gene—encoding the metabotropic glutamate receptor mGluR6—have been identified as the mutated gene in X-linked recessive and autosomal recessive complete CSNB [8-10], respectively. Both nyctalopin and mGluR6 proteins are distributed on the postsynaptic ON BCs and are required for the depolarization of the cell. The *NYX* gene appears to be the major, and possibly only, causative gene for X-linked recessive complete CSNB since *NYX* gene mutations were identified in the majority of X-linked recessive families with complete CSNB. In contrast, *GRM6* gene mutations have been found in only some of the autosomal recessive families with complete CSNB, indicating the existence of other unknown genes for autosomal recessive complete CSNB.

We previously identified a mouse transient receptor potential cation channel, subfamily M, member 1 (*Trpm1)* homolog of human *TRPM1* [11]. *Trpm1* is alternatively spliced, resulting in the production of a long form protein (trpm1-L) and a short N-terminal form devoid of transmembrane segments (trpm1-S). Although mouse trpm1-S was previously identified as melastatin [12], mouse trpm1-L has not been identified. Without distinction between trpm1-L or trpm1-S, trpm1 has been reported to be detected in human primary melanocytes [13], poorly metastatic melanoma cell lines [14,15], mouse retinal pigment epithelium (RPE) [16], and subsets of ON and hyperpolarization of neuron by light (OFF) BCs [17,18]. We found that trpm1-L localization is developmentally restricted to the dendritic tips of ON BCs in co-localization with mGluR6 [11,19]. *Trpm1* null mutant mice completely lose the photoresponse of ON BCs. Trpm1-L channel activity is negatively regulated by activated G_o_ in the mGluR6 cascade, and we showed that Trpm1-L is a component of the ON BC transduction channel. Interestingly, the *trpm1*-deficient mice showed similar electrophysiological responses to the responses of those with complete CSNB—significantly reduced b-waves—which led us to examine whether the gene is mutated in human patients with complete CSNB.

Very recently three groups reported the association of TRPM1 mutations with CSNB [20-22]. Independently from these studies, in our present study, we identified five different novel mutations in the human *TRPM1* gene: IVS2–3C>G, IVS8+3_6delAAGT, R624C (c.1870C>T), S882X (c.2645C>A), and F1075S (c.3224T>C). In addition, our biochemical and cell biologic analyses suggested that the two intron mutations were likely to result in abnormal protein production by abnormal splicing, and the two missense mutations lead to the mislocalization of the TRPM1 protein in BCs. Fundus examination revealed no abnormalities other than myopic changes, and the single bright-flash, mixed rod-cone ERG showed a “negative-type” configuration with a reduced normal a-wave and a significantly reduced b-wave amplitude.

## Methods

### Subjects

Four separate patients with complete CSNB (#204, #373, #437, and #484) in whom previous molecular examination revealed no mutation in either *NYX* or *GRM6* among 11 separate patients were analyzed. They were all male, and their ages were 26, 9,19, and 27 years, respectively. They complained of night blindness from early childhood, and otherwise had no health problems. All individuals examined had been under observation at the Department of Ophthalmology of Nagoya University, Nagoya, Japan. The research protocol was designed in compliance with the Declaration of Helsinki and approved by the institutional review board. Written informed consent was obtained from the subjects after an explanation of the purpose of this study was provided. Briefly, the protocol entails molecular analysis of causative genes from blood samples of patients with complete CSNB and in vitro analysis including expression assay and cytological assay using the samples without any disadvantages for the patients.

### DNA analysis of patients with complete CSNB

Genomic DNA was extracted from the peripheral blood leukocytes. Exons 2 through 27 of *TRPM1* were amplified by polymerase chain reaction (PCR) using the DNA Thermal Cycler 9700 (Applied Biosystems, Foster City, CA). Primers were designed by and purchased from Invitrogen (Carlsbad, CA). The primer sets used in this study are shown in Table 1. For all exons, 100 ng of genomic DNA was amplified in a 50 µl reaction with 0.5 µM of each primer, 0.2 mM of each dNTP, and Ampli*Taq* Gold polymerase (Applied Biosystems). The PCR conditions were as follows: 5 min at 94 °C; 35 cycles at 94 °C for 30 s, followed by 30 s at 54 °C, and 45 s at 72 °C; and a final extension step at 72 °C for 7 min. The PCR products were directly sequenced using an ABI PRISM 3100 Genetic Analyzer (Applied Biosystems) and BigDye Terminator v3.1 Cycle Sequencing Kit (Applied Biosystems). To search for polymorphisms, exons 3, 8, 16, 21, and 24 of *TRPM1* from 200 alleles (50 men and 50 women) from unrelated, normal Japanese individuals were directly sequenced.

**Table 1:** The primer sets used in this study.

**Exon**	**Direction**	**Primer sequence (5′-3′)**
2	Forward	GCTTGGGAATGTATTGGTCC
2	Reverse	TTCTGGACTCCTCTTTCTGC
3–1	Forward	TTGCTAATGAAGGGGAAAGG
3–1	Reverse	TGAAAGCTGTGATCCTAGTG
4–2	Forward	TACCCAACAGATTCCTATGG
4–2	Reverse	TTGTGGATTCAGAGCTTTGG
4	Forward	TGCTTGGACATGAGCAATAG
4	Reverse	TATTAGGAAGCTCTTTGGGG
5	Forward	GATCTGTAGGGAATGTAGGG
5	Reverse	CAACTGCATCTGAATCAACG
6	Forward	TATTGTGCTTGCTTCCCTTG
6	Reverse	GCAATCAATCTCCTTCTCAG
7	Forward	TACTCTTCCCCATACTTCAG
7	Reverse	CGTTTGGAATGTCTAAGCAG
8	Forward	CCACAGCAAAGTCTCAAATC
8	Reverse	AATAACCCTGTCATGCACAC
9	Forward	CAGTCTCTGTGGTCATTTTG
9	Reverse	TTCATGCTGAGACTGCTCAAG
10	Forward	AAGCCAGCAATGCTTAGTTC
10	Reverse	TGTCACAAGGGAGAACATAC
11	Forward	ATGTGTGCACATACAGAGGT
11	Reverse	GAAGCAAGGACAAGAACCAT
12	Forward	ACCCTTTCCTCTGTGTTTCC
12	Reverse	GCACAATATGCACCAGTGAC
13	Forward	TACTGACCTCAAGTGATCTG
13	Reverse	TTCATTATCTCCTGTGCCTG
14	Forward	CTGTTACATGAACTCCCAAG
14	Reverse	TTGTTGAAAAGCAAGCGAGG
15	Forward	CCATTCGTATGTCCACTATC
15	Reverse	GAGTAGTATCGTATATTCGC
16	Forward	AGTTCTGGAACTCTTTGTTG
16	Reverse	AGAGACAAGTACTCAGGATG
17	Forward	GGTGGATATGCCTGTCTAAG
17	Reverse	AAAGTGCTCAGTTCAGCCTG
18	Forward	GGCTCCTAGGTTATTGAATA
18	Reverse	GTGGTACAAGAAATCTCAAC
19	Forward	TTCTAATGGTGCCTGTGTGC
19	Reverse	CAATGTTAGCCAGAGATCTC
20	Forward	GTGTCAACCTGAAAGAAAAC
20	Reverse	TTTCAGTAAGGAGCCATATC
21	Forward	ATGACCTGGAATGTTCCATC
21	Reverse	GCCTGTTCTTATGTCCTAAC
22	Forward	TATAATGAGCCAGGGTGAAC
22	Reverse	TTTGGAACTGATCATCTGCC
23	Forward	CTTTTCTCTCAACCATCATC
23	Reverse	ACAGTGAATTTGTGGTTCTC
24	Forward	AGCTGGGGCTACAGAGTTTA
24	Reverse	TATCTTGGGAGCGTTCTGAG
25	Forward	GTAACTTTGACTGCTCTGGG
25	Reverse	CGAGCAAGTAGTTGAGTGAG
26	Forward	GGGCTGTGAAATTCTCATTG
26	Reverse	AACTCTGGCATCCCCCATAG
27–1	Forward	CATGTTAGGGGATTGTGAAG
27–1	Reverse	ATACGTTGCCTCACATTCAG
27–2	Forward	CAGAATGGTGAATGCTCTTG
27–2	Reverse	GAAAGGTGAAGACTGTTCTG
27–3	Forward	AAAAAAACCTGTTCCTTCCG
27–3	Reverse	TTTGTCGTTTCCACTGTTAG
27–4	Forward	GAAGAAACTATTTCCCCAAG
27–4	Reverse	GAATATCTGTGCTATGAGAG
27–5	Forward	TAGAGTACAGTTCAATCACG
27–5	Reverse	ATGGATTTCACATTCCTAGG
27–6	Forward	TCTGTGAAGCCAGATCAAAC
27–6	Reverse	GTTTAGATGGCCAAGATGAC

### *TRPM1* splicing minigene constructs

DNA fragments of *TRPM1* exon 2 with its downstream splice donor region (nucleotide position in GenBank # NC_000015: 24738–25055; exon 2+SD) and exon 8 with its downstream splice donor region (39020–39392; exon 8+SD) were PCR-amplified using human genomic DNA as a PCR template; SacI and KpnI sites were appended to the 5′ and 3′ ends, respectively. A partial fragment of β-globin (*HBB*) intron 2 (614–1166 in GenBank # NC_000011) was PCR-amplified; KpnI and SalI sites were appended to the 5′ and 3′ ends, respectively. DNA fragments of *TRPM1* exon 3 with its upstream splice acceptor region (31325–31691; SA+exon 3) and exon 9 with its splice acceptor region (39976–40280; SA+exon 9) were PCR-amplified using human genomic DNA as a PCR template; SalI and BamHI sites were appended to the 5′ and 3′ ends, respectively. The exon 2+SD, *HBB* intron, and SA+exon 3 fragments (exon 2+3 construct), or exon 8+SD, *HBB* intron, and SA+exon 9 (exon 8+9 construct) were ligated and subcloned into the SacI and BamHI sites of *pBluescript KS+* (Stratagene), containing three repeats of the Flag-tag between the BamHI and NotI sites. The generated exon 2+3 construct and exon 8+9 construct were digested with SacI and NotI and subcloned into SacI and BamHI digested pEGFP-C3 and pEGFP-C2 (Clontech, Palo Alto, CA), respectively, each with a synthesized BglII-NotI linker.

### Transfection and western blots analysis

HEK293T cells were cultured in D-MEM containing 10% fetal bovine serum (FBS; Nissui, Tokyo, Japan). These cells were grown under 5% carbon dioxide at 37 °C. Transfections were performed using the calcium phosphate method. Transfected cells were incubated at 37 °C for 48 h, and harvested for further analysis. Cell extract proteins were separated by SDS–PAGE, and transferred to a polyvinylidene difluoride membrane (ATTO, Tokyo Japan). The membrane was incubated with a mouse anti-Flag antibody (1:1,000; Sigma, St Louis, MO), a rabbit anti-β−gal antibody (1:10,000; Chemicon, Temecula, CA), or a mouse anti-β−actin antibody (1:2,000; Sigma), and incubated with a horseradish peroxidase-conjugated goat anti-mouse or -rabbit IgG (1:10,000; Zymed Laboratories, San Francisco, CA). The bands were visually developed using Chemi-Lumi One L (Nacalai Tesque, Kyoto, Japan).

### In vivo electroporation and section immunohistochemistry

In vivo electroporation was performed at postnatal day 0 (P0) as described previously [23]. Approximately 0.3 μl of DNA solution (5 μg/μl) was injected into the subretinal space of P0 mouse pups, and square electric pulses (80 V; five 50 ms pulses with 950 ms intervals) were applied with electrodes. Wild-type (WT) and mutant forms of *TRPM1* (R624C and F1075S) containing 3× Flag at the C-terminus were expressed from the mouse *mGluR6* promoter (mGluR6-TRPM1–3×Flag). To generate this construct, the chicken β-actin gene (CAG) promoter region in *CAG-TRPM1* was replaced with the 9.5 kb fragment of *mGluR6* 5′ upstream genomic sequence [24]. mGluR6-TRPM1–3×Flag (4 μg/μl) and CAG-GFP (1 μg/μl) were co-electroporated and harvested at P14. Immunohistochemistry to observe the subcellular localization of TRPM1–3×Flag expressed in the electroporated ON BCs was performed on sections as previously described [23]. Mouse anti-Flag antibody (1:500), rabbit anti-PKCα antibody (1:20,000; Sigma), and rat anti-GFP antibody (1:1,000; Nacalai Tesque) were used. The signal intensity of Flag immunostaining at the dendritic tips and somas of ON BCs was measured using AxioVision software (Carl Zeiss, Oberkochen, Germany). The ON BCs that showed more intensity in the dendritic tips than in the somas were counted. One hundred electroporated ON BCs were counted from three retinas independently prepared for each construct.

### Clinical evaluations in patients associated with *TRPM1* mutations

The ophthalmologic examination included best-corrected visual acuity, refraction, biomicroscopy, ophthalmoscopy, fundus photography, Goldmann kinetic perimetry, color vision testing, and electroretinography [25]. Standardized full-field ERGs were elicited by Ganzfeld stimuli after pupillary dilation with 0.5% tropicamide and 0.5% phenylephrine hydrochloride and 20 min of dark-adaptation. The rod (scotopic) ERGs were elicited by a blue stimulus with a luminance of 5.2×10^−3^ cd/sec•m^2^. The mixed rod-cone, single flash (bright white) ERGs were elicited by a white stimulus of 44.2 cd/sec•m^2^. The photopic (cones) single-flash ERGs and 30 Hz flicker ERGs were elicited by white stimuli at a luminance of 4 cd/sec•m^2^ and 0.9 cd/sec•m^2^, respectively, on a white background of 68 cd/m^2^. The single flash bright white ERGs were recorded again after 17 h of dark adaptation.

### ERG recordings in the *trpm1* null mutant (*trpm1^–/–^*) mice

We generated *trpm1****^–/–^*** mice as described previously [11]. Our methods for recording the ERGs from the mice have been described in detail [26]. In brief, the mice were dark-adapted overnight, and then anesthetized with an intramuscular injection of 70 mg/kg ketamine and 14 mg/kg xylazine. ERGs were recorded with a gold-wire loop electrode placed on the anesthetized cornea. The mice were placed in a Ganzfeld bowl and stimulated with stroboscopic stimuli of 1.0 log cd-s/m^2^ (photopic units) maximum intensity. Four levels of stimulus intensity ranging from −5.0 to 1.0 log cd-s/m^2^ were used for the dark-adapted ERG recordings, and four levels of stimulus intensity ranging from −0.5 to 1.0 log cd-s/m^2^ were used for the light-adapted ERGs. The light-adapted ERGs were recorded on a rod-suppressing white background of 1.3 log cd-s/m^2^ (photopic units). The amplitude of the a-wave was measured from the baseline to a set time of 8 msec after the initial negative wave for the dark-adapted ERG, and 11 msec for the light-adapted ERG. The b-wave amplitude was measured from the negative trough of the a-wave to the maximal positive peak.

### Animal care

All procedures conformed to the ARVO Statement for the Use of Animals in Ophthalmic and Vision Research, and these procedures were approved by the Institutional Safety Committee on Recombinant DNA Experiments and the Animal Research Committee of Osaka Bioscience Institute. The mice were housed in a temperature-controlled room with a 12h:12h light-dark cycle. Fresh water and rodent diet were available at all times.

## Results

### DNA analysis of the complete CSNB patients

**Figure 1:**
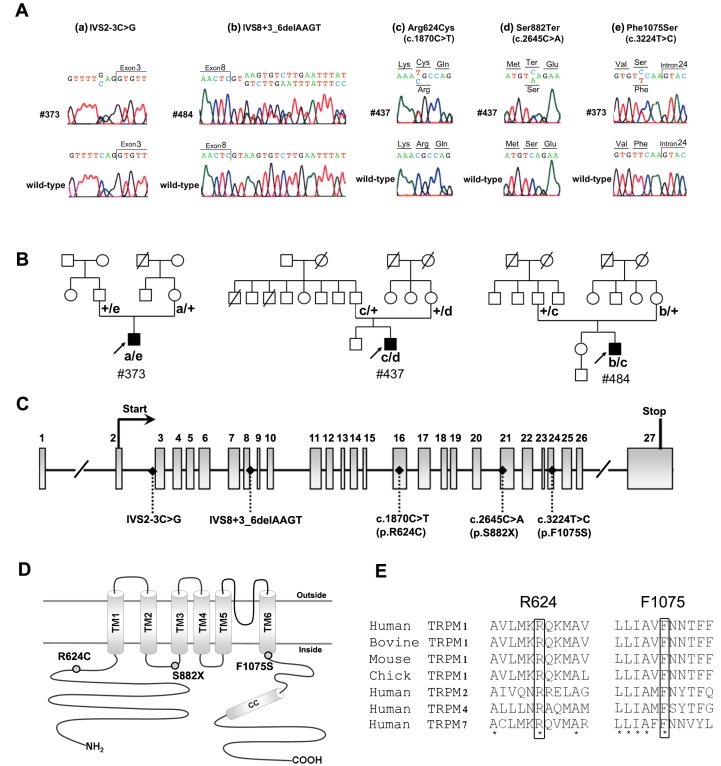
Compound heterozygous *TRPM1* mutations identified in patients #373, #437, and #484. **A**: Sequence chromatograms showing the mutations: IVS2–3C>G in patient #373 (a), IVS8+3_6delAAGT in patient #484 (b), Arg624Cys (c. 1870C>T) in patients #437 and #484 (c), Ser882Ter (c. 2645C>A) in patient #437 (d), and Phe1075Ser (c. 3224T>C) in patient #373(e). **B**: Complete CSNB pedigrees of Japanese patients #373, #437, and #484. These three patients are compound heterozygotes of *TRPM1* mutations. **C**: Exon structures of human *TRPM1*. The first methionine (Start) and a stop codon (Stop) of the *TRPM1* open reading frame are indicated. All mutations found in this study are shown. **D**: Putative topology of the human TRPM1. All mutations found in this study are illustrated. The six transmembrane domains are indicated as TM1-TM6. **E**: Alignment of R624 and F1075 in TRPM proteins. Sequence alignment of TRPM1 from human, bovine, mouse, chick, and TRPM2, TRPM4, and TRPM7 from human. Amino acid residues R624 and F1075 are boxed. The asterisks indicate completely conserved residues.

Five different mutations in *TRPM1* were identified: IVS2–3C>G, IVS8+3_6delAAGT, R624C (c.1870C>T), S882X (c.2645C>A), and F1075S (c.3224T>C). Mutations were present in three unrelated patients with complete CSNB (#373, #437, and #484). All three patients were compound heterozygotes. Their pedigrees and the DNA sequence chromatogram for the patients harboring these mutations are presented in Figure 1A,B.

The two intron changes, IVS2–3C>G and IVS8+3_6delAAGT (Figure 1C), were interpreted to be pathogenic because no RNA splicing activities were detected when each of the two changes was present (see splicing assay section). They were also predicted to affect RNA splicing, as assessed by an online splice-site prediction tool. The donor site prediction score from the software for the exon 8 donor site in *TRPM1* was 0.99 for the WT sequence, whereas it was under 0.01 for the corresponding mutated sequence with the IVS8+3_6delAAGT sequence change. The acceptor site prediction score for the exon 3 acceptor site in *TRPM1* was 0.98 for the WT sequence, whereas it was 0.44 for the mutated sequence with the IVS2–3C>G sequence change. The two mutations were not found in an analysis of 100 Japanese normal controls.

The S882X mutation was considered to be a pathogenic null mutation since mRNA containing the predicted early stop codon would most likely be subject to nonsense mediated decay without being translated into a protein [27]. Even if the translated protein was produced, it would lack the C-terminus region of TRPM1, including transmembrane domains 4–6, which is very likely to lead to a loss of function as a cation channel (Figure 1D). This change was not found among the 100 healthy control individuals we examined. The two missense changes in the *TRPM1* gene, R624C and F1075S, were located in the N-terminal region and in the C-terminal end of the transmembrane domain, respectively (Figure 1D). They were interpreted as likely pathogenic because the arginine at position 624 and the phenylalanine at 1075 are well conserved among the TRPM subfamily, suggesting the importance of these amino acid residues for TRPM function (Figure 1E). The two mutations were not found in an analysis of 100 Japanese normal controls. None of the five mutations mentioned above have been reported in a SNP database.

### Analysis of protein expression from *TRPM1* mutant minigenes

**Figure 2:**
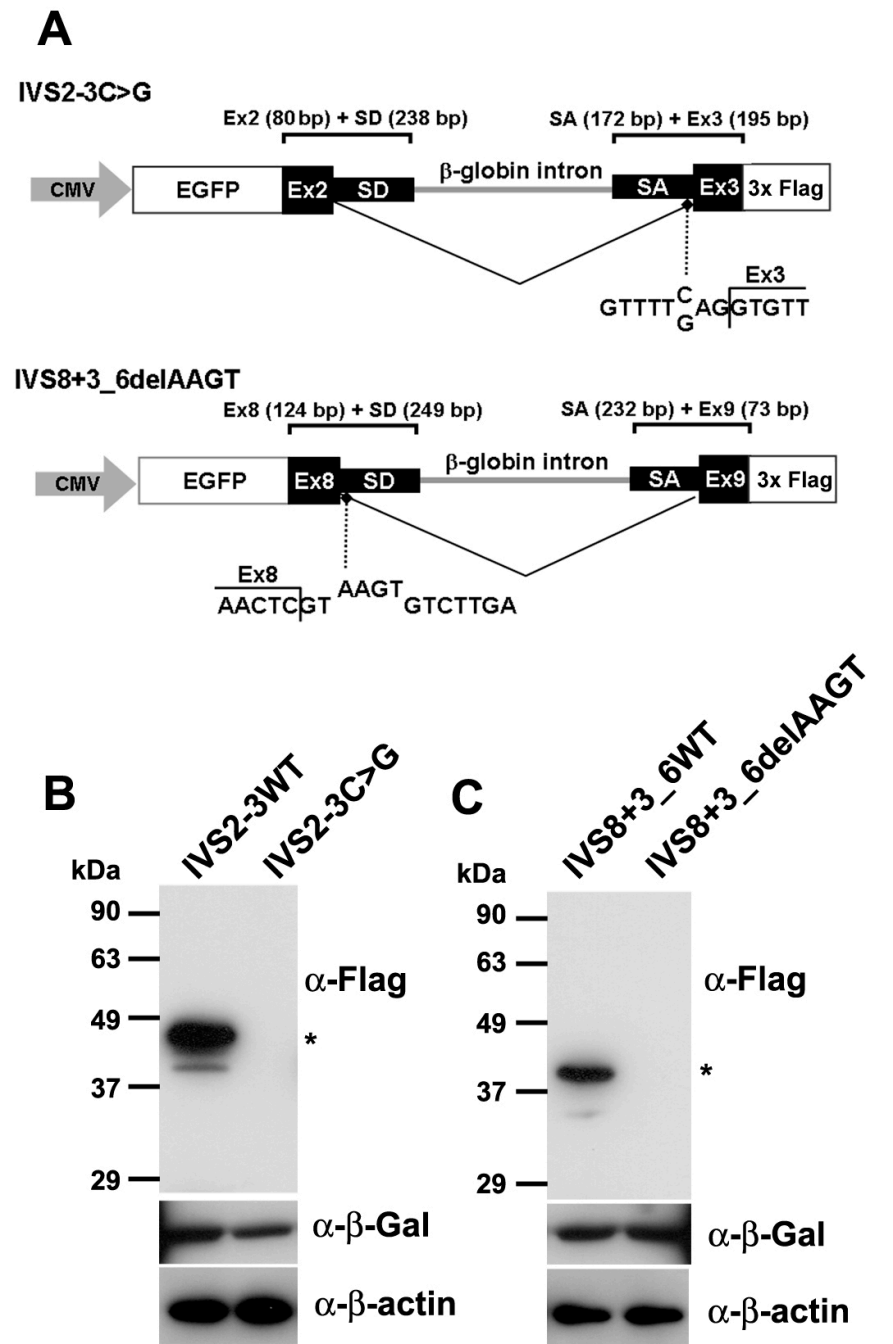
Analysis of protein expression from *TRPM1* mutant minigenes. **A**: Schematic of *TRPM1* minigenes. The minigene *IVS2–3C>G* contains an *EGFP* gene, exon 2, the exon 2 splice donor region, a portion of the β-globin (*HBB*) intron 2, the exon 3 splice acceptor region, and exon 3, and uses the *CMV* promoter and *SV40 polyA* site from the *pEGFP-C3* vector. The minigene IVS8+3_6delAAGT contains an *EGFP* gene, exon 8, the exon 8 splice donor region, a portion of the *HBB* intron 2, the exon 9 splice acceptor region, and exon 9, and uses the CMV promoter and *SV40 polyA* site from the *pEGFP-C2* vector. Mutations of IVS2–3C>G and IVS8+3_6delAAGT are shown. **B**-**C**: western blots of HEK293 cell extracts transfected with minigenes IVS2–3C>G (**B**) or IVS8+3_6delAAGT (**C**). WT represents protein from a wild-type minigene transfection, and asterisks represent spliced EGFP detected by an anti-Flag antibody. β-Gal and β-actin were used for transfection control and loading control, respectively.

Our previous expression analysis of mouse TRPM1 showed that the long form (TRPM1-L), which functions as a cation channel, is predominantly expressed in the retina [11], suggesting that human patient samples for directly analyzing splicing are not readily available. To overcome this limitation, minigenes for expression in tissue culture cells were designed; these minigenes included the upstream exons (exon 2 or 8) fused with EGFP, downstream exons (exon 3 or 9) fused with 3×Flag-tags, and a *HBB* intron, which is often used in splicing assays (an exon 2+3 minigene and an exon 8+9 minigene; Figure 2A). Since the signals required for constitutive splicing are usually within ~100 nucleotides of splice junctions [11], we took ~200 nucleotide regions for both splicing donors and acceptors to ensure the splicing assay. We also generated mutated minigenes containing the IVS2–3C>G mutation in the exon 2+3 minigene or the IVS8+3_6delAAGT mutation in the exon 8+9 minigene. Minigene splicing was analyzed in HEK293T cells since they have been used extensively to study constitutive RNA splicing. HEK293T cells were transfected with minigene DNA and a lacZ expression internal control vector, and western blotting was performed on whole cell extracts isolated 48 h later. We then analyzed protein products of the EGFP-exon 2+3 fusion and the EGFP-exon 8+9 fusion proteins by western blot analysis of whole cell extracts transfected with the WT and mutated minigenes. We detected an expected 45 kDa or 39 kDa band with the anti-Flag antibody in HEK293T cell extracts transfected with the WT exon 2+3 minigene or the exon 8+9 minigene (Figure 2B,C). In contrast, we did not detect any bands in either of the cell extracts transfected with the mutated minigenes. We detected the β-gal bands of the transfection internal control, and the β-actin bands of the cell extract control in both the WT and mutant minigene transfected cells. These results showed that both the IVS2–3C>G and IVS8+3_6delAAGT mutations abrogate normal splicing and lead to abnormal protein production, suggesting that these *trpm1* alleles are loss-of-function alleles.

### In vivo analysis of intracellular localization of TRPM1 protein carrying missense mutations

**Figure 3:**
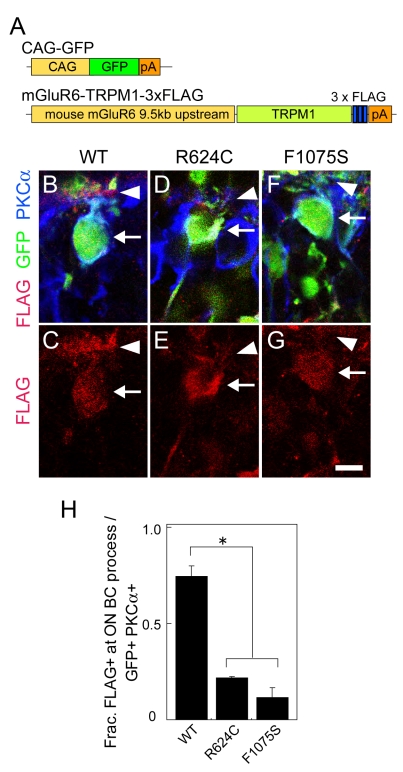
In vivo electroporation of WT, R624C, and F1075S *TRPM1* expression vectors fused with the *mGluR6* promoter. **A**: DNA constructs used for electroporation. *CAG-GFP* was co-electroporated to identify electroporated regions. WT and mutant forms of *TRPM1* fused with *3×Flag* were expressed under the mouse *mGluR6* 9.5 kb promoter. **B**-**G**: Section immunohistochemistry of the P14 retinas electroporated in vivo at P0 with *CAG-GFP* and *mGluR6-TRPM1–3×Flag.* The sections were immunostained with PKCα (blue), GFP (green), and Flag (red) antibodies (**B**, **D**, and **F**). Immunostaining with Flag was visualized separately (**C, E** and **G**). Arrows represent the soma and arrowheads represent the dendritic tips of ON BC. Scale bar: 10 μm. **H.** Composition of electroporated ON BC (GFP+ PKCα+), where bright Flag signal was observed in the dendrites. The error bars represent standard deviation (SD). Asterisks show that the differences are statistically significant (n=3) (Student’s *t*-test, p<0.05).

The TRPM1 missense mutations R624C and F1075S are located in the N-terminal and the C-terminal end of the six-transmembrane domains (6TM), respectively (Figure 1D). Mutations in these regions often cause misfolding/mislocalization of the protein, and/or a defect in channel activity [28-32]. To determine the functional relevance of R624C and F1075S TRPM1 residues, we mutagenized the *TRPM1* cDNA to introduce R624C and F1075S mutations. We first prepared expression vectors encoding R624C or F1075S mutant proteins attached with a Flag-tag at the C-terminus, and expressed them both, as well as WT vectors, in HEK293T cells. We did not observe a significant difference in the protein level between the mutants and WT by western blotting, suggesting that the protein stability of R624C and F1075S was not affected (data not shown). We then prepared expression vectors of *R624C-* and *F1075S-TRPM1* cDNAs fused with the mouse *mGluR6* 9.5 kb promoter that drives ON BC-specific expression (Figure 3A). We electroporated each of them as well as the WT vector into the P0 mouse retinas. We examined the localization of the expressed TRPM1 mutant proteins in ON BCs at P14 when the endogenous mouse TRPM1 is preferentially localized to the dendritic tips [11,19]. The electroporated human TRPM1 was visualized by an anti-Flag tag antibody. TRPM1 WT was localized at the soma (arrow) and dendritic tips (arrowheads) of the ON BCs (Figure 3B,C). On the other hand, the immunostaining signals of both the R624C and the F1075S TRPM1 proteins were significantly weaker than those of the WT protein (Figure 3D-G). We quantified the number of electroporated ON BCs in which the WT TRPM1 protein was localized more in the dendritic tips than somas. About 70% of ON BCs that electroporated with WT TRPM1 showed a brighter TRPM1 signal at the dendritic tips, and the signals on the dendritic tips were dramatically reduced in both mutant proteins (Figure 3H). These results may imply that R624C and F1075S mutations lead to mislocalization of TRPM1 protein and are responsible for the protein trafficking of TRPM1 channel proteins. However, it should be noted that there is also a possibility that the transfected TRPM1 proteins are misfolded due to the addition of the 3xFlag tag, leading to the undetectability of Flag-tagged mutant proteins specifically in the dendrites. Future analysis is required to obtain a conclusive result on this point.

### Clinical characteristics of the complete CSNB patients associated with *TRPM1* mutations

**Figure 4:**
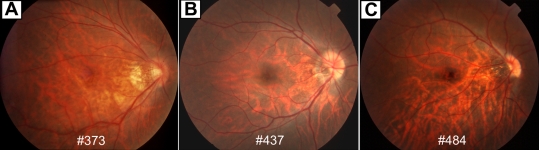
Fundus photographs of patients with mutations of *TRPM1*. **A-C**: Fundus images of patient #373 (**A**), patient #437 (**B**), and patient #484 (**C**). All fundus examinations revealed no abnormalities in the posterior pole other than myopic changes, including the tilted discs and temporal pallor of the optic discs. The patient’s number is indicated in each photograph.

The clinical characteristics of the three complete CSNB patients with *TRPM1* mutations are summarized in Table 2. All the patients showed the typical clinical features of complete CSNB. They complained of night blindness from their early childhood, and the best-corrected visual acuities were normal in two patients (#373 and #437) and mildly reduced in one (#484). The refractive errors were highly or moderately myopic, and astigmatism was present in all of the patients (Table 2). Mild nystagmus and esotropia was observed in the patient with reduced visual acuity (#484). Slit lamp examination revealed no abnormality, and all fundus examinations revealed no abnormalities in the posterior pole other than myopic changes, including the tilted discs and temporal pallor of the optic discs (Figure 4). Goldmann kinetic visual fields and color vision tests with the Ishihara plates, the AO H-R-R pseudoisochromatic plates, and Farnsworth D-15 panel test were conducted in two patients (#437 and #484) with normal results. No family history was obtained from the patients (Figure 1B).

**Table 2:** Clinical Characteristics in complete CSNB with TRPM1 mutations.

**Mutation**	**Corrected visual acuity**	**Refraction**	**Color vision**	**Fundus appearance**	**Visual field**	**Night blindness**	**Nystagmus**	**Strabismus**
**patient #373 (Male, 9 years old)**								
IVS3–3C>G, F1075S	1.0 (OD)	−8.25 −2.75 X 40 (OD)	ND	Myopic	ND	+	−	Orthophoria
	1.0 (OS)	−8.25 −0.50 X 180 (OS)						
**patient #437 (Male, 19 years old)**								
R624C, S882X	1.0 (OD)	−4.50 −2.25 X 120 (OD)	Normal	Myopic	Normal	+	−	Orthophoria
	1.0 (OS)	−4.50 −2.75 X 50 (OS)						
**patient #484 (Male, 27 years old)**								
IVS9+3_6delAAGT, R624C	0.5 (OD)	−13.00 −3.00 X 90 (OD)	Normal	Myopic	Normal	+	+	Esotropia

**Figure 5:**
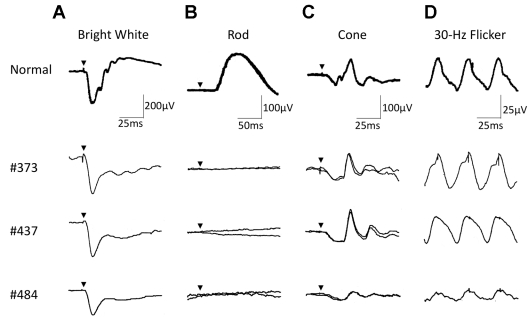
Full-field ERGs of patients with mutations of the *TRPM1* gene. **A**-**D**: Full-field ERGs recorded in a normal subject and three affected individuals. The single bright-flash, mixed rod-cone ERGs showed a “negative-type” configuration with a reduced normal a-wave and a significantly reduced b-wave amplitude (**A**). The scotopic ERGs showed no response after 20 min of dark-adaptation (**B**). The photopic ERGs showed an apparent a-wave with normal implicit time and a b-wave with delayed implicit time (**C**). The amplitudes of the 30 Hz flicker ERGs were within normal range (**D**). The oscillatory potentials were reduced. Arrowheads: stimulus onset. Each patient’s number is noted at left.

The full-field scotopic (rod) ERGs elicited by a blue light were non-recordable after 20 min of dark-adaptation (Figure 5B). The photopic ERG showed an a-wave with apparently normal implicit time and a b-wave with delayed implicit time (Figure 5C). The amplitudes of the 30 Hz flicker ERG were within normal range (Figure 5D). The single bright-flash, mixed rod-cone ERG elicited by a white stimulus had a “negative-type” configuration with a reduced normal a-wave and with a significantly reduced b-wave amplitude (Figure 5A). The oscillatory potentials were reduced.

### ERG analysis of the *trpm1* null mutant (*trpm1^–/–^*) mice

**Figure 6:**
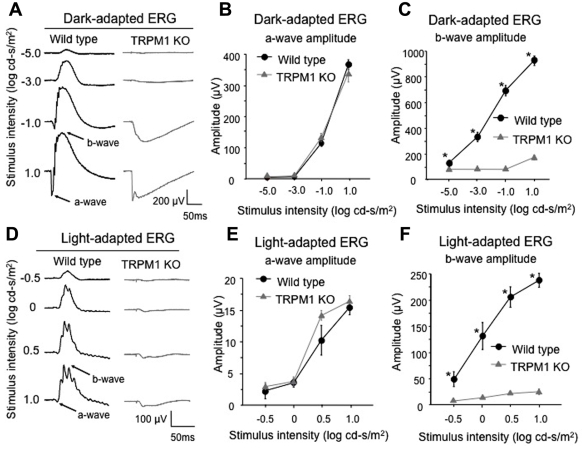
ERG of WT and *trpm1^–/–^* mice. **A**-**F**: ERGs recorded from 8-week-old mice. Dark-adapted (**A)** and light-adapted (**D**) ERGs were elicited by four different stimulus intensities in both WT and *trpm1^–/–^* mice (n=5). Amplitudes of dark-adapted (**B**) and light-adapted (**E**) ERG a-waves as a function of the stimulus intensity are shown. Amplitudes of dark-adapted (**C)** and light-adapted (**F**) ERG b-waves as a function of the stimulus intensity are shown. The bars represent the standard error of the mean (SEM). Asterisks show that the differences are statistically significant (Mann–Whitney test, p<0.05). The *trpm1^–/–^* mouse had a normal a-wave, but a severely depressed b-wave for both dark- and light-adapted ERGs.

To address a possible role of TRPM1 in ON BC function, we previously generated *trpm1* null mutant (*trpm1^–/–^*) mice by targeted gene disruption [11]. Next, to compare the physiologic retinal function of *trpm1^–/–^* between humans and mice, we recorded mouse ERGs in 8-week-old mice (Figure 6). The dark-adapted (rod) ERGs elicited by different stimulus intensities from WT and *trpm1^–/–^* mice are shown in Figure 6A. The amplitudes of the a-wave, originating from the photoreceptors, were approximately equal for both types of mice (Figure 6B). In contrast, the dark-adapted ERG b-wave, originating mainly from the ON BCs, in *trpm1^–/–^* mice was not present at the lower intensities, and only a very small positive inflection was seen at the highest stimulus intensity of 1.0 log cd-s/m^2^ (Figure 6C). The ERGs elicited by different stimulus intensities from light-adapted WT and *trpm1^–/–^* mice are shown in Figure 6D. The b-wave was severely depressed or absent leaving only the a-wave in the *trpm1^–/–^* mouse (Figure 6E,F). These ERG results are similar to those obtained by other groups [19,33], and suggested that the function of both rod and cone ON BCs, and the signal transmission from rod and cone photoreceptors to BCs were severely impaired in *trpm1^–/–^* mice. In contrast, the presence of a normal a-wave indicated that the rod and cone photoreceptors were functioning normally in this mutant mouse.

## Discussion

An essential step in intricate visual processing is the segregation of visual signals into ON and OFF pathways by retinal BCs [34]. The release of glutamate from photoreceptors modulates the photoresponse of ON BCs [35] via metabotropic glutamate receptor 6 (mGluR6) and the G-protein (G_o_) that regulates a cation channel [36-38]. We recently reported that we identified a mouse *trpm1* long form (*trpm1-L*) and found that TRPM1-L localization is developmentally restricted to the dendritic tips of ON BCs in co-localization with mGluR6 [11]. *Trpm1* null mutant mice completely lose the photoresponse of ON BCs. TRPM1-L channel activity is negatively regulated by activated G_o_ in the mGluR6 cascade. These results suggest that TRPM1-L is a component of the ON BC transduction channel. These observations led us to examine whether the gene is mutated in human patients with complete CSNB in the current study.

To identify human *TRPM1* gene mutations in CSNB patients, we analyzed four separate Japanese patients with complete CSNB in whom previous molecular examination revealed no mutation in either the *NYX* or *GRM6* genes. In the current study, we identified five different mutations in *TRPM1*: IVS2–3C>G, IVS8+3_6delAAGT, R624C (c.1870C>T), S882X (c.2645C>A), and F1075S (c.3224T>C). Mutations were present in three unrelated patients with complete CSNB (#373, #437, and #484). Using a minigene expression assay, we showed that two intron mutations, IVS2–3C> and IVS8+3_6delAAGT, can cause splicing abnormalities leading to defects in protein production. Since these two mutations are located in the N-terminus region of TRPM1, these mutations are likely to produce a loss-of-function allele of *TRPM1*. The nonsense mutation S882X (c.2645C>A) is located between TM2 and TM3. Thus, the truncated TRPM1 protein is likely to be non-functional as a channel. Regarding the two missense mutations, R624C (c.1870C>T) and F1075S (c.3224T>C), we observed failure of the transportation of the missense mutant channels to the dendritic tips. However, ~20% of ON BCs could transport the mutant forms of TRPM1, suggesting that these ON BCs are still active. Considering that human patients carrying these mutations still have cone ERG responses, this fraction might be cone ON BCs converging inputs from cone photoreceptors. Since these two amino acid residues are evolutionarily conserved among the TRPM subfamily, these amino acid residues are indispensable for the physiologic functions of TRPM channels. For example, R671Q mutation in the *Drosophila TRPM1* homolog (*trp^14^*) resulted in a significant reduction of ERG response [39]. This mutation is located between the 6TM and the TRP domain [40], which is close to the F1075 residue of human TRPM1. Although this region is not in the functional domains, it still could be responsible for the physiologic function of the TRPM channel.

The scotopic ERG b-waves recorded using Ganzfeld stimuli were nonrecordable, and the photopic ERG showed normal a-wave amplitude, but the amplitude of the b-wave is reduced or absent in the three affected human individuals. There seems to be no apparent genotype-phenotype correlation in our patients with *TRPM1* mutations. Full-field ERG results showed no detectable post-receptoral ON-pathway function for the three patients. The ERG amplitudes of patient #484 were smaller than those of the other two patients, but the reductions were considered to be due to high myopia (−12 to −13 D) [41] or other unknown reasons. The function of the inner retina was further analyzed using L-M cone ERGs elicited by rectangular, 100–125 msec duration light stimuli under light-adapted conditions in one patient, and as a result, the amplitude of the b-wave at light onset (ON-response) was significantly reduced, but the d-wave amplitude (OFF-response) at light offset was unaffected with normal OPs on the OFF-responses (data not shown). These electrophysiological results indicated that the pathology in complete CSNB with *TRPM1* mutations lay in the dysfunction of the depolarizing ON BCs because it is generally considered that the positive ON-response (b-wave) reflects the depolarizing ON BCs. The impairment in the ON-response was also observed in the *trpm1****^–/–^*** mice in which b-wave amplitude for both scotopic and photopic conditions were completely defective. These results in human and mice strongly indicated that TRPM1 is essential for the function of ON BCs in the visual pathway.

The clinical features of patients with complete CSNB are similar even when the causative gene is different. Most patients complain of night blindness while showing normal fundi accompanied by highly myopic refractive errors [4,5]. The best-corrected visual acuity is mildly reduced, or sometimes normal. We could not clarify any difference in ERG responses that were recorded according to ISCEV protocol among those with different gene defects. To date, the only phenotypic difference among complete CSNB patients with different gene mutations has been detected using a scotopic 15 Hz-flicker ERG with increasing intensities [42]. It was reported that patients with *TRPM1* mutations showed 15 Hz-flicker ERG responses similar to those with *NYX* mutations, but different from those with *GRM6* mutations or normal control subjects, suggesting some difference in the rod pathway [21].

Very recent studies reported that *TRPM1* gene mutations were the major cause of AR complete CSNB in patients with Caucasian ancestors [20-22]. We previously identified *NYX* mutations in five families and *GRM6* mutations in two families among 11 Japanese families with complete CSNB [43,44], and in the current study, we found *TRPM1* mutations in three of the remaining four families. From these results we confirmed that the gene was responsible for patients with complete CSNB. It is likely that one of the three genes that is localized at the dendritic terminals of ON BCs and contributing the cell activity, *TRPM1*, *GRM6*, or *NYX*, would responsible for most patients with complete CSNB.

In conclusion, we identified five different mutations in the *TRPM1* gene in three unrelated Japanese patients with complete CSNB. *TRPM1* is essential for the depolarizing ON BC function in humans as well as in mice.
